# Explosive Growth of the Jorō Spider (*Trichonephila clavata* (L. Koch): Araneae: Araneidae) and Concurrent Decline of Native Orbweaving Spiders in Atlanta, Georgia Forests at the Forefront of the Jorō Spider’s Invasive Spread

**DOI:** 10.3390/insects16050443

**Published:** 2025-04-23

**Authors:** Robert W. Pemberton

**Affiliations:** Independent Researcher, 2275 1st Ave NE, Atlanta, GA 30317, USA; rpemberton5@gmail.com

**Keywords:** Araneidae, census, native species, population change, predation

## Abstract

The Jorō spider (*Trichonephila clavata* (L. Koch): Araneae: Araneidae), an invasive species from Asia, and native orbweaving spiders were censused at 25 forest sites in the Atlanta, Georgia, region at the forefront of the Jorō spider’s spread from 2022 through 2024. The Jorō spider was found in all 25 sites in all three years, doubling in abundance each year. In 2022, the number of Jorō spiders found was 444 or 16.34 per hour of census. This doubled in 2023 to 859 or 30.54 per hour. The Jorō numbers doubled again in 2024 to 1713 or 59.14 per hour. This contrasts markedly with the seven species of native orbweavers found during the censuses, which declined by ca. 40% each year. In 2022, 52 or 1.72 individuals per hour of six native orbweaver species were found at 18 sites. In 2023, the number dropped to 32 or 1.06 individuals per hour of six species found at 11 sites. In 2024, the number further declined to 18 or 0.55 individuals of six species found at eight sites. The Jorō spider’s spread and rapid increase in abundance may disrupt trophic networks and negatively influence the abundance of both native orbweavers and flying insect species.

## Simple Summary

The Asian Jorō spider was found in northeast Georgia in 2014, and the forefront of the spider’s westward spread reached Atlanta in 2022. The sudden arrival of large numbers of Jorō spiders presented an opportunity to determine the abundance and monitor changes in the spider and the native orbweaving spiders that might be impacted by it. The Jorō spider and the native orbweavers were censused in 25 forests in the Atlanta region from 2022 through 2024. The Jorō was found in all 25 sites in all three years, doubling in abundance each year. In 2022, the number of Jorō spiders found was 444 or 16.34 per hour of census, which grew in 2024 to 1713, or 59.14 per hour. This contrasts markedly with the seven species of native orbweavers found during the censuses, which declined by ca. 40% each year. In 2022, 52 or 1.76 individuals per hour of six native orbweaver species were found at 18 sites. By 2024, the number dropped to 18 or 0.55 individuals per hour of six species found at eight sites. The Jorō spider’s spread and rapid increase in abundance may disrupt food webs and negatively influence the abundance of both native orbweavers and flying insect species.

## 1. Introduction

### 1.1. Established Non-Native Spiders

Alien invasive species are one of the main drivers of biodiversity decline and the reduction of ecosystem services worldwide [1,2]. The number of invasive species is increasing and impacting the world’s ecosystems and biodiversity, resulting in economic consequences [3,4]. Although spiders are among the many types of animals that naturalize and invade, they have received less attention and research than plants and insects [5,6]. When numerous communities were inventoried, almost 25% had at least one established non-native spider species l [7]. Global trade of commodities is thought to be responsible for most alien spider introductions [8]. Spiders may be well suited to being shipped as stowaways in commercial shipping containers because of their ability to survive without food for long periods [9]. Established non-native spider species are generally thought to be synanthropic [10], hence the many alien building and house spiders [11].

### 1.2. Jorō Spider Introduction to North America

The Asian Jorō spider was first found in North America in northeastern Georgia, USA, in 2014 [12], although it may have been present as early as 2010 [13]. It probably arrived in commercial shipments of goods [12] from Japan, Korea, or China. It has since spread to adjacent states, including Tennessee, the Carolinas, and Alabama [14,15]. It is likely spreading by ballooning in the juvenile stage, as is common in orbweavers, for regional spread within Georgia and the adjacent states. The spiders are also probably being spread by human transport activities, as indicated by the discovery of Jorō spiders well beyond their primary range in the Georgia–Tennessee–Carolinas area. They occur in West Virginia, Maryland, and during the fall of 2024, they were found in coastal Virginia, as well as in Philadelphia, Pennsylvania, and Boston, Massachusetts in the northeastern United States, and in Florida in the southern United States [15]. Davis and Frick [16] found that the Jorō spider has a higher metabolism and is more cold- tolerant than its congener *Trichonephila clavipes* (L. Koch), whose distribution within the United States is limited to the southeastern states. This led to the prediction that it has the potential to spread much farther north, potentially to the northeast, which is now occurring. Recent modeling of the Jorō spider’s potential distribution in North America, using its known occurrences in its native Asia, predicted that it could spread to the Great Lakes region [16]. Giulian et al. [17] constructed a Maximum Entropy Model to predict the areas of North America that are climatically suitable for the Jorō spider. The model found a land area of 2,566,547 km^2^ that was above 50% suitability for the spider, which would be a 20-fold increase from the 120,000 km^2^ recently estimated to be the area of occupation in 2022 [13]. The Jorō spider’s spread may be similar to the spread of many established non-native insects, which is characterized by occasional long-distance dispersal, resulting in colonies that grow, coalesce, and greatly increase spread [18].

An important recent survey of the Jorō spider and native orbweavers in northern Georgia found this invasive spider to be the most abundant species at just over half of the 103 surveyed sites [14]. Their large, 1 m plus wide webs and the high densities of these webs in invaded forests suggest significant capture and predation of flying insects. DNA metabarcoding of prey remains in Jorō spider webs, fecal samples, and their dissected gut contents found that this spider has a broad diet, with Diptera being the most frequent prey type [19,20]. The spider’s large size, abundance, frequent occurrence in human habitats [21], and rapid geographic spread has alarmed the public, a perspective which is due, in part, to the news media’s sensationalism and portrayal of the spider as a threat to people [13].

While the Jorō spider had been spreading from northeastern Georgia since it was first found in 2014, it was only in 2022 that the spider became abundant in Atlanta. It had been detected in the two Atlanta counties of Fulton in 2018 and Dekalb in 2020 but was sparse. Prior to 2022, there were only four iNaturalist postings of the Jorō spider for Dekalb County [22]. This grew to 108 postings in 2022, 344 in 2023, and 570 in 2024. In 2021, I noted none in my forested residence but in 2022, when the spider became common and even abundant, I counted 32 Jorō spiders in my 0.12 ha forested residence. The sudden appearance of the many Jorō spiders presented an opportunity to document its occurrence and abundance at the forefront of its invasive spread into Atlanta forests. It also enabled the documentation of the presence and abundance of other orbweaving spiders that the Jorō spider might impact. The goals of this study were to detect the presence and measure the abundance of the Jorō spiders and native orbweavers in Atlanta forests during three years of censusing to detect potential change in each. This study found that the presence of Jorō spiders doubled with each year while the number of native orbweavers declined ca. 40% each year. The decline in native orbweavers may be related to the great and increasing abundance of the Jorō spiders, perhaps through shared predation on flying insects. The Jorō spiders spread and increasing abundance may disrupt trophic networks and may negatively influence the abundance of both native orbweavers and flying insect species.

## 2. Materials and Methods

### 2.1. Study Sites and Data Collection

Jorō spiders and other aerial orbweaving spiders were censused in 25 Atlanta, Georgia forests in 2022, 2023, and 2024. The censusing was done once a year at each site during September and October when female spiders and their webs were large and more apparent. The method was similar to the Pollard walk used to count orbweavers by Lubin [23] and Jorō spiders in Japan by Miyashita [24]. The numbers of female Jorō spiders and other orbweavers were counted along paths and the areas ca. 5 m from the paths and along forest margins, during timed daytime censuses, which were usually about one hour but varied somewhat with the size of the site. The censusing at each site was done on the same date plus or minus 3 days each year, and along the same walking route. Because the amount of time censusing at each site varied somewhat each year, the number of spiders reported was calculated as the number per hour per site each year.

During 2024, the number of Jorō spider webs without females was counted to obtain information about possible predation of the spiders. The identifications of the orbweavers other than the Jorō spider were made with Bradley [25], Rose [26], and iNaturalist research-grade postings of orbweavers in Georgia (https://www.inaturalist.org/). Most (22/25) of the forests were located inside the ring road (Interstate 285) that circles Atlanta and nearby municipalities in Fulton and Dekalb counties at the forefront of the Jorō spider’s westward spread and population expansion. These forest sites were in an area of ca. 250 km^2^ (within 33.91° N, −84.23° E, 33.67° S, and −84.38° W). This area is in the eastern half of the Atlanta ring road (Interstate 285), bounded on the north by I 285, on the east by I 285, on the south by I 285, and on the northwest by US 19, and the southwest by US 23. All of the forests were separate, distinct units within or surrounded by residential or other urban developments. The three sites outside the ring road were one site (Chattahoochee NP) in Fulton County, ca. 10 km to the north; one site (Panola) DeKalb County, ca. 10 km southeast of the ring road; and another (Little Mulberry) in Gwinnett County, adjacent to DeKalb County, ca. 40 km to the east. Table S1 with the GPS positions of the sites is available. All sites were located in forests in nature preserves and nature parks, with the exception of the forested residence of the author. All sites, except the author’s, were open to the public and easily accessible. The forests are secondary but with mature old growth with large trees, including some champion trees of their species for the area. These are hardwood forests comprised principally of native *Quercus*, *Acer*, and *Carya* species, along with *Betula nigra* L., *Liriodendron tulipifera* L., *Magnolia grandiflora* L., and some *Pinus taeda* L. The understory included a varied mix of native and exotic shrubs, while the ground level was frequently dominated by non-native English ivy (*Hedera helix* L.) and non-native grass-like lily turfs (*Liriope* spp.).

### 2.2. Statistical Analysis

To compare the number of Jorō spiders vs. native orbweaving spiders recorded per hour, a generalized linear mixed model (GLMM) was used with *proc glimmix* and the log link function-mixed model. A repeated measures analysis of variance was conducted using SAS ver. 9.4 [27]. Here, sites were used as random variables, and years were the repeat variable. A Sidak-protected LSMeans was performed to determine differences between spiders counted per hour during each year (*p* = 0.05).

## 3. Results

### 3.1. Jorō Spider Presence and Abundance

**Figure 1:**
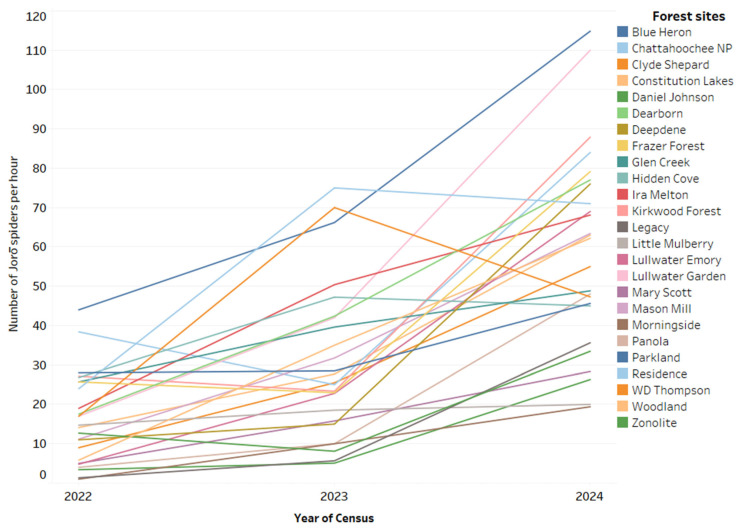
Number of Jorō spiders found per hour at 25 forests sites in Atlanta, Georgia, during three years of censusing, emphasizing the change at each site.

**Figure 2:**
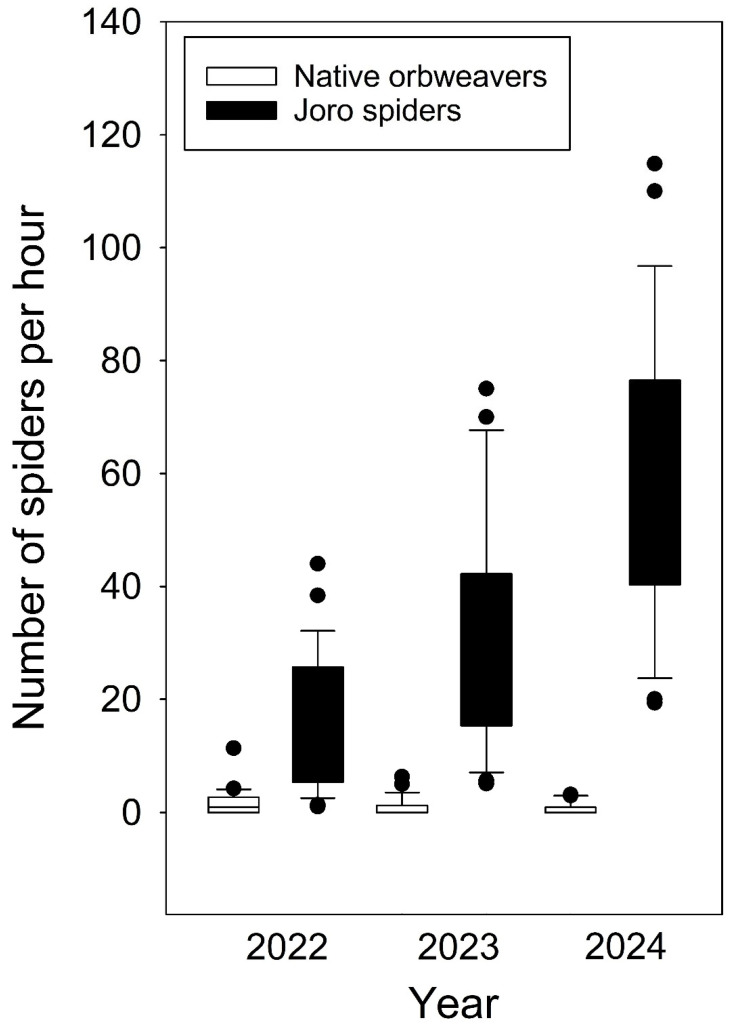
Box plot of spiders found per hour, showing both Jorō spiders and native orbweavers.

The number of Jorō spiders found at each site in the 25 forests surveyed in 2022, 2023, and 2024 is shown in Table 1. Changes in the numbers of Jorō spiders at each site in each year are illustrated in Figure 1. The number of Jorō spiders increased significantly from 2022 to 2023 (t_72_ = 4.50; *p* < 0.0001) and from 2023 to 2024 (t_72_ = 4.68; *p* < 0.0001) (Figure 2. In 2022, the spider was found at all the forest sites, with a total of 444 individual Jorō spiders found with a mean number of 18.86 per hour per site (Table 1). The numbers per site varied considerably from relatively few individuals (only one spider at two sites and less than 10 per site at seven sites) to sites with many Jorō spiders (more than 30 individuals at four sites, and more than 40 at two sites). The number of Jorō spiders found per hour per site in 2022 was 17.8. In 2023, the Jorō spider was found at all sites and the total number doubled from 444 to 859 (Table 1), with a mean of 34.36 per site. Only two sites had fewer than 10 Jorō spiders and 17 sites had more than 30, while five sites had more than 50, and three sites had more than 70. In 2024, Jorō spiders were found again at all sites, with the total number doubling from 859 in 2023 to 1761, with 68.52 per site. All sites had more than 10 spiders, and only two had fewer than 20, while 17 had more than 50, 11 had more than 70, and five had more than 100. The number of Jorō spiders found per hour per site in 2024, at 59.14, was about three times the 16.34 per hour found in 2022. From 2022 to 2023, Jorō spiders increased at 21 of the 25 sites and decreased slightly at four. From 2023 to 2024, the number of Jorō spiders per hour increased at 23 of the 25 sites and decreased at two, and the number of Jorō spiders per hour from 2022 to 2024 increased at all sites. Figure 2 shows box plots of the highly significant changes in Jorō spider abundance.

### 3.2. Native Orbweaver Presence and Abundance

**Figure 3:**
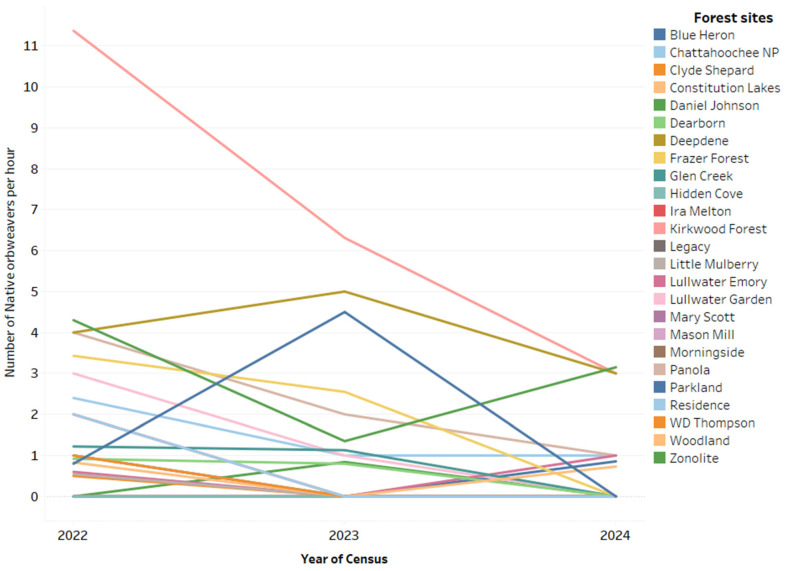
Number of native orbweavers found per hour at 25 forest sites in Atlanta, Georgia, during three years of censusing, emphasizing the change at each site.

Seven species of native orbweavers were found (Table 2). The changes in the numbers of native orbweavers at each site in each year are illustrated in Figure 3. In 2022, six species of native orbweavers were found at a total of 18 of the 25 sites. A total of 52 individual spiders were found, with a mean number of 1.96 per site and 1.72 per hour. In 2023, six species were found at 11 sites, and the number of individuals declined to 32, with an average of 1.28 per site and 1.06 per hour. In 2024, 18 individuals of six species were found at eight sites. The mean number per site was 0.72 and 0.55 per hour. The decline in number of native orbweavers was significant between 2022 and 2023 (t_72_ = 2.02; *p* = 0.0475), and between 2022 and 2024 (t_72_ = 3.26.; *p* = 0.0017) (Figure 2). There was no difference in native orbweaver numbers between 2023 and 2024 (t_72_ = 1.36; *p* = 0.1770).

The closely related *T. clavipes* (Golden silk orbweaver) was found only during the first year of census in 2022, when four individuals were found at three sites. The most abundant native orbweaver found was *Gasteracantha cancriformis* (Linnaeus, 1758) (Spinybacked orbweaver), followed by *Araneus marmoreus* Clerck, 1757 (Marbled orbweaver) (Table 2).

### 3.3. Jorō Spider Versus Native Orbweaver Abundance

During the three years of censusing, the Jorō spiders doubled each year while the native orbweavers declined by 40% each year. In 2022, there were about nine Jorō spiders for every native orbweaver (444/52 = 8.55:1). In 2023, there were 27 Jorō spiders for every native orbweaver (859/32 = 26.8:1), and in 2024, there were almost 100 Jorō spiders for every native orbweaver (1713/18 = 95.16:1). In just two years—from the first year of the Jorō spider’s sudden, significant appearance in Atlanta in 2022 to the third season in 2024—the relative abundance of Jorō spiders compared to native orbweavers increased by more than tenfold. The two-way interaction between spider species and years was significant (F _2,72_ = 24.05; *p* < 0.0001) (Figure 2). More Jorō spiders were found per hour than native orbweavers during 2022 (t_72_ = 10.51; *p* < 0.0001), 2023 (t_72_ = 14.29; *p* < 0.0001), and 2024 (t_72_ = 16.33; *p* < 0.0001) (Figure 2).

## 4. Discussion

### 4.1. The Findings

The number of Jorō spiders at the sites was, at times, probably larger than the numbers indicate. At some sites, such as the Chattahoochee Nature Center, Jorō spider webs were removed along paths by the staff to make the paths more comfortable for visitors prior to the census (Jacquiline McRae pers. com). Similar removal of Jorō spider webs and spiders is suspected at other more-visited forest sites, such as the Woodland site. In 2024, when the number of empty Jorō spider webs was recorded at each site, empty webs increased after a cold period on 17 October, when temperatures dropped to 1.7 °C (https://www.weather.gov/ffc/October2024ClimateSummary) (accessed 5 January 2025). Before this date, empty webs were usually torn and often had male Jorō spiders still present, suggesting that the missing female Jorō spiders were perhaps victims of predation by birds [28]. After the cold period, the empty Jorō spider webs were more often intact and males were absent, suggesting that females had abandoned their webs to ascend trees to lay eggs. At the last two census dates of the season, there were more empty webs than occupied webs (29 October at Hidden Cove: 51 empty webs versus 39 webs with Jorō spiders; 30 October at Glen Creek: 62 empty webs versus 27 webs with Jorō spiders). This likely indicates that the population of Jorō spiders was significantly higher than the number of females remaining in the webs indicated.

The rather dramatic increase of the Jorō spider and the concurrent decline of the native orbweavers may be due to the indirect effect of greater predation of available flying insect prey by the Jorō spider and their occupation of web construction sites. The observed Jorō spider webs were much larger than all the other orbweavers except for the congener *T. clavipes*, which was relatively rare and encountered only during the first year. Jorō spider webs, at times, spanned 1 to 2 m and, with their associated barrier webs, often occupied considerable areas. The Jorō spider webs can also be dense. For instance, at one site (the forest residence) in 2024, there were ca. 80 webs in an area of 0.12 ha. Furthermore, these webs often were spread across paths and creeks in these forests that appear to function as flyways for many insects. The Jorō spider is active both diurnally and nocturnally [13], which allows it to capture both insects that are active in both time periods. Nocturnally active orbweavers that have larger, heavier, and higher webs, such as the Jorō spider’s, are known to catch heavier insect prey [29], which provide more calories, resulting in larger females, with higher fecundity [20].

The dramatic increase in Jorō spiders and the concurrent decline in native orbweavers is not proof that the Jorō spider caused the decline of native orbweavers. There are no data on the abundance and diversity of native orbweavers in these forests or other Atlanta region forests prior to the Jorō spider’s arrival. Other unknown factors may be responsible for the decline in the abundance of native orbweavers, but their simultaneous decline during the explosive increase of Jorō spider’s suggest that the Jorō spider influenced their decline.

### 4.2. Finding Compared to the Prior Study

This censusing study of Jorō spider and other native orbweavers is only the second study to quantify their presence, and the first study to examine their numbers over time to detect changes in each. It is also the first study to quantify the Jorō spider’s abundance at the forefront of their invasive spread. The first and only other study was done by Nelsen et al. 2023 [14] in northern Georgia in 2022 in a single year. In this census, Jorō spiders and 19 native orbweaver species were counted for 10 min after encountering the first spider along 103 transects. They found the Jorō spider at just over half of these sites (53/103 or 51.5%), with a total of 543 Jorō spiders and an average of 5.7 Jorō spiders per site. One native orbweaver, *Micrathena mitrata* (Hentz, 1850), was the second most abundant spider (319), found at almost half of the sites (49/103 or 47.6%) and averaging more than three individuals per site. Another species, *Neoscona crucifera* (Lucas, 1838), was observed at >40% of surveyed locations (44 of 103 or 42.7%) but averaged less than one per site. All other spiders had fewer than one individual per site. The Atlanta forest census found Jorō spiders at all 25 sites in all three years, with an average number of Jorō spiders of 18 per site in 2022, which rose to 34 in 2023, and 68 in 2024. The numbers of Jorō spiders in the Nelsen et al. study and in this study cannot easily be compared because the total time or average time per site was not given in the Nelsen et al. study, but it was apparently much less than the average time per site of about 70 min in the Atlanta study. A more meaningful comparison, however, can be made by examining the relative differences between the numbers of individual Jorō spiders and native orb weavers. The Nelsen et al. study found somewhat more individual native orbweavers (754) than individual Jorō spiders (543), a ratio of ca 1.4 native orbweavers to each Jorō spider. This contrasts markedly with the present study, where in the first year of the census, there were ca. 10 Jorō spiders to every native orbweaver, which grew to ca. 100 Jorō spiders to one native orbweaver in 2024. The Nelsen et al. study found more native orbweavers—19 versus 7 species in this study—and a much greater abundance of these spiders than was found in the Atlanta census. This greater number of native species in the Nelsen et al. study may be due to the much greater number of sites (103 vs. 25) and the larger geographic area that the study surveyed. It is interesting to note that *M. mitrata*, the most abundant orbweaver after the Jorō in the Nelsen et al. study, was not detected in the Atlanta census. The Nelsen et al. study found fewer native orbweaver species at sites where the Jorō spiders were known to occur prior to their 2022 surveys, suggesting possible displacements of the native orbweavers by the Jorō spiders. The Nelsen et al. study suggested that the Jorō spider may be more tolerant of higher human populations than the native orbweavers.

### 4.3. Potential Impacts of the Jorō Spider

#### 4.3.1. On Native Orbweavers

Chuang et al. [13] listed native orbweavers in the Southeastern United States that they thought would be most likely to be impacted by the Jorō spider. Among the 14 species listed, seven were found in this study’s census of Atlanta forests (Table 2). As mentioned above, the Jorō spider congener, *T. clavipes*, was only found during the first year of censusing. This spider is more common south of the Atlanta area in the coastal areas of the Southeastern United States [26]. Of these seven native orbweavers found in the Atlanta study, four species declined during the three years, while one species had the same numbers, and two slightly increased. Except for the overall decline in the native orbweavers, the numbers of these spiders were so low that the year-to-year changes in these species may not be meaningful. The larger species listed by Chuang et al. [13]—*Argiope aurantia* (Lucas, 1833) and *Argiope trifasciata* (Forsskål, 1775)—were not detected in the Atlanta study. These species tend to occur in more open habitats than in forests where the Atlanta censuses were conducted. *Araneus bicentenarius* (McCook, 1888) is uncommon [26]. A recent study by Grabarczyk et al. [20] examining the diets of both female and male Jorō spiders using barcode analysis enabled the identification of 109 unique taxa from 57 families of consumed arthropod prey. Two families of spiders (Araneidae and Philodromidae) were identified, indicating that Jorō spiders predate other orbweavers (Araneidae).

Studies on the potential competition between coexisting orbweavers may provide insights into the potential competition between Jorō spiders and native orbweavers. Horton and Wise [30] manipulated two *Argiope* spider species to examine interspecific competition and found only minor influence on the growth and survival of these spiders. Two other studies (Richardson and Hanks [31] and Novak et al., 2010 [32]) found that partitioning of available prey allowed the sympatric spiders to coexist.

#### 4.3.2. On Native Insects

Of the many insect prey types that the Jorō spider takes, bees and wasps were [33] found to be the most abundant prey in their Jorō spider web surveys. I have also observed many Hymenopteran pollinators in Jorō spider webs, including honeybees, bumblebee species, and hornets. Hymenoptera are among the most beneficial insect groups because bees are among, if not the most important, pollinators, and predatory and parasitic wasps are important regulators of insect populations. This Davis et al. Jorō spider web prey survey differs from what was found by the Grabarczyk et al. [19] metabarcoding analysis of Jorō spider prey types. The study’s seemingly contradictory findings were that Diptera were the most abundant in prey remains in Jorō spider webs and fecal pellets, while Coleoptera were the most abundant prey order in female gut contents. Nelsen et al. [14] observed *T. clavata* feeding on prey in several different arthropod groups, including cockroaches, beetles, wasps, bees, butterflies, dragonflies, grasshoppers, and other spiders. The aforementioned Grabarczyk et al. [20] study found that Diptera, especially the Chironomidae, were the most common prey type of this spider. The study identified 55 families of insects and 109 unique arthropod taxa. The high metabolic rate and quick maturation time of the Jorō spider [16] suggest that it consumes a large amount of prey as well as a great diversity of prey. This reduction or loss of prey for native orbweavers may account for their observed decline in this study. Jorō spider predation of insect pests could be beneficial, especially forest pest insects, but research is needed to begin to understand the consequences of their predatory behavior on insects and possible influences on trophic networks.

### 4.4. Documented Impacts of Other Established Non-Native Spiders on Native Spiders and Insects

There is very limited literature on the impacts of established non-native spiders on either native spiders or native insects. The invasive *Linyphia triangularis* (Clerck) led to reduced densities of native spiders [34,35], increased web abandonment and web building of the native *Frontinella communis* Hentz, and web takeovers of *F. communis* in Maine, United States [36]. The candy-striped spiders (*Enoplognatha ovata* (Clerck) and *E. latimana* Hippa and Osala) are known to disproportionately prey on pollinators, even actively hunting prey during pre-dawn periods when insects are typically lethargic [37].

The Australian redback spiders (*Latrodectus hasseltii* Thorell) are invasive, opportunistic predators that threatens New Zealand fauna, especially the endangered chafer beetle [38]. Invasive species are among the important causes of the loss of biodiversity, including insects [39]. Established non-native spiders interfere with the local community and guild structure because of predation of insects and other spiders [6].

### 4.5. Jorō Spider Natural Enemies

The great abundance of Jorō spiders may be a product of natural enemy release [40] in the invaded area. In a study of two widow spiders (*Lactodectus* spp.), one invasive and the other native, the invasive spider suffered less parasitism by specialist egg parasitoids both in nature and in the laboratory [41]. Spiders in the genus *Nephila* (closely related to *Trichonephila*) are reported to experience relatively infrequent predation [42]. Almost nothing is known about the natural enemies in its native Asia. In Japan, part of the Jorō spider’s native region, it is occasionally attacked by an ectoparasitic ichneumonid wasp [43]. The only reported insect natural enemy of the Jorō in North America are mud-daubing wasps (Hymenoptera: Sphecidae), which provisioned their brood cells with the juvenile Jorō spiders (Hoebeke and L.A. Taylor, cited in Chuang et al. [13]). It appears that birds are predators of Jorō spiders in the United States, as first suggested by Schronce and Davis [44], when a Northern Cardinal (*Cardinalis cardinalis* L.) was observed to lunge at a Jorō spider. This bird species was subsequently observed to attempt to predate on caged Jorō spiders and repeatedly attacked a tethered Jorō spider [28]. Damaged orb webs without a spider present is strong evidence of a predator attack [45]. The Northern Cardinal is among the most predaceous of common birds [46]. This bird and/or other insectivorous birds may be responsible for the many destroyed Jorō spider webs encountered during the Atlanta censuses. However, at least to date, predation by sphecid wasps and birds appears to have limited impact on Jorō spider abundance. Research is ongoing in both the invaded area (Georgia) and the native region (Japan and Korea) to detect potential parasites and predators of Jorō spider eggs (Pemberton, unpublished). Some invasive species develop explosive populations after becoming established, but their populations subsequently subside as local natural enemies learn and or adapt to use them [47,48]. It is too early in the Jorō spider’s North American invasion to guess about its long-term population trends. Biological control, through the introduction of co-evolved host-specific natural enemies, has been used to control invasive species [49], but suitable natural enemies may not exist or, if available, may be ineffective. If the Jorō spider’s environmental impacts become as severe as this study suggests they could, it could be worthwhile to consider establishing a biological control project against it. Science, however, has barely begun to understand the ecology and potential environmental impacts of the Jorō spider in North America.

## Figures and Tables

**Table 1:** Number of Jorō spiders and native orbweavers in 25 forest sites in Atlanta, Georgia during a 3-year census in 2022, 2023, and 2024.

.	2022						2023						2024					
D = Dekalb, F = Fulton,	Date	Time	Jorō	Native	Jorō	Native OW ^1^	Date	Time	Jorō	Native	Jorō	Native OW ^1^	Date	Time	Jorō	Native	Jorō	Native OW ^1^
G = Gwinnett				OW ^1^	per hour	per hour				OW ^1^	per hour	per hour				OW ^1^	per hour	per hour
Residence (D)	25-September	50	32	2	38.4	2.4	25-September	60	25	1	25	1	24-September	60	84	1	84	1
Parkland (D)	23-September	60	44	1	44	1	25-September	68	75	0	66	0	25-September	70	134	1	114.85	0.86
Deepdene (D)	24-September	60	11	4	11	4	25-September	60	15	5	15	5	25-September	60	76	3	76	3
Legacy (D)	25-September	45	1	0	1.33	0	26-September	53	5	0	5.66	0	25-September	64	38	0	35.62	0
Daniel Johnson (D)	26-September	85	18	6	12.7	4.3	27-September	89	12	2	8.09	1.35	29-September	95	53	5	33.47	3.15
Lullwater Garden (D)	26-September	60	17	3	17	3	28-September	60	42	1	42	1	27-September	60	110	0	110	0
Frazer Forest (D)	26-September	35	15	2	25.71	3.43	27-September	47	18	2	22.97	2.55	27-September	47	62	0	79.14	0
Kirkwood Forest (D)	27-September	95	43	18	27.15	11.37	29-September	95	37	10	23.36	6.31	30-September	101	148	5	87.9.	3
Panola (D)	14-October	60	4	4	4	4	15-October	60	10	2	10	2	14-October	60	48	1	48	1
Woodland (D)	14-October	68	16	0	14.11	0	15-October	65	30	0	27.69	0	14-October	60	63	0	63	0
Little Mulberry (G)	15-October	110	27	1	14.72	0.545	14-October	123	38	0	18.53	0	15-October	120	40	0	20	0
WD Thompson (D)	16-October	120	18	1	9	0.5	16-October	120	51	0	25.5	0	18-October	120	110	0	55	0
Lullwater Emory (D)	16-October	100	8	1	4.8	0.6	17-October	100	38	0	22.8	0	16-October	60	69	1	69	1
Chattahoochee (F)	17-October	60	24	2	24	2	18-October	60	75	0	75	0	17-October	60	71	0	71	0
Clyde Shepard (D)	18-October	60	17	1	17	1	19-October	60	70	0	70	0	18-October	80	63	0	47.3	0
Mason Mill (D)	22-October	102	19	0	11.17	0	22-October	85	45	0	31.76	0	22-October	88	93	0	63.4	0
Ira Melton (D)	23-October	60	19	2	19	2	23-October	50	42	0	50.4	0	23-October	52	59	0	68.1	0
Constitution Lakes (D)	23-October	72	7	1	5.83	0.83	24-October	72	42	0	35	0	23-October	82	85	1	62.19	0.73
Dearborn (D)	24-October	65	19	1	17.54	0.92	24-October	75	53	1	42.4	0.8	24-October	74	95	0	77.02	0
Blue Heron (F)	26-October	75	35	1	28	0.8	26-October	80	38	6	28.5	2.5	25-October	96	73	0	45.62	0
Zonolite (D)	27-October	71	4	0	3.3	0	27-October	71	6	1	5.07	0.84	26-October	64	28	0	26.3	0
Mary Scott (D)	27-October	36	3	0	5	0	27-October	38	10	0	15.79	0	26-October	36	17	0	28.33	0
Morningside (F)	28-October	60	1	0	1	0	28-October	60	10	0	10	0	29-October	62	20	0	19.4	0
Glen Creek (D)	28-October	49	21	1	25.71	1.22	28-October	53	35	1	39.62	1.13	30-October	43	35	0	48.83	0
Hidden Cove (D)	29-October	47	21	0	26.81	0	29-October	47	37	0	47.23	0	29-October	52	39	0	45	0
Totals		1705	444	52	408.38	43.92		1751	859	32	763.57	26.48		1766	1713	18	1478.47	13.74
Average		68.2	17.8/site	1.96/site	16.34/h	1.76/h		70.04	34.36/site	1.28/site	30.54/h	1.06/h		70.02	68.52/site	0.72/site	59.14/h	0.55/h
Mean		68.2	17.76	2.08	16.34/h	1.72		70.04	34.36	1.16	30.54/h	1.06/h		70.64	68.52	0.72	61.23	0.56
SD		22.4	11.91	3.64	11.44	2.44		21.74	20.65	2.21	19.70	1.76		25.51	33.66	1.46	26.22	1.01

^1^ OW = orbweavers.

**Table 2:** Orbweaving spiders found during the censusing of 25 forest sites in Atlanta, Georgia, and the numbers found each year.

	2022	2023	2024
1. *Trichonephila clavata* (Jorō spider) (11–32 mm ^1^), diurnal and nocturnal ^1^	444 at 25 sites	859 at 25 sites	1752 at 25 sites
2. *Trichonephila clavipes* (Golden silk orbweaver) (19–34 mm), diurnal and nocturnal	4 at 3 sites	None	None
3. *Araneus marmoreus* (Marbled orbweaver) (9–18 mm), nocturnal	1 at 1 site	10 at 5 sites	3 at 3 sites
4. *Gasteracantha cancriformis* (Spinybacked orbweaver) (5.8–8.6 mm), diurnal	19 at 5 sites	11 at 3 sites	3 at 1 site
5. *Neoscona crucifera* (Cross orbweaver) (8.5–19.7 mm), nocturnal	9 at 6 sites	2 at 2 sites	1 at 1 site
6. *Micrathena gracilis* (Spined micrathena) (7–11 mm), diurnal	None	1/1 site	2 at 1 site
7. *Verrucosa arenata* (Triangulate orbweaver) (5–9.5 mm), nocturnal	5 at 3 sites	8 at 5 sites	2 at 2 sites
Total number of individuals	52	32	18
Number of sites	18	11	8
Number of species	6	6	6

^1^ Spider sizes and activity periods are for females from Rose (2022) [26], except for *Neoscona crucifera*, which is from Bradley (2013) [25].

## Data Availability

The source data are provided in Table 1.

## Supplementary Materials

The following supporting information can be downloaded at: https://www.mdpi.com/article/10.3390/insects16050443/s1.
