# Supplementary material for: Explosive Growth of the Jorō Spider (Trichonephila clavata (L. Koch): Araneae: Araneidae) and Concurrent Decline of Native Orbweaving Spiders in Atlanta, Georgia Forests at the Forefront of the Jorō Spider’s Invasive Spread
Source: Insects. 2025 Apr 23;16(5):443. doi: 10.3390/insects16050443 (PMC12112690; doi:10.3390/insects16050443)
Supplement: Supplementary file 1 [file insects-16-00443-s001.zip › insects-3466773-supplementary.pdf]

Supplementary Table S1. Locations of Atlanta, Georgia forest sites where the Jorō spider and native orbweavers were censused (2022, 2023, 2024).

Forest Sites	Location	Latitude, Longitude
Blue Heron Nature Preserve	Fulton County	33.863823, -84.377211
Chattahotchee Nature Center	Fulton County	34.004088, -84.382791
Clyde Shepherd	Dekalb County	33.807032, -84.283027
Constitution Lakes Park	Dekalb County	33.6829681, -84.344741
Daniel Johnson Nature Preserve	Dekalb County	33.797485, -84.341478
Dearborn Wildlife Preserve	Dekalb County	33.757367, -84.287302
Deepdene Park	Atlanta	33.772469, -84.320755
Frazer Center Forest	Atlanta	33.770222, -84.327838
Glen Creek Nature Preserve	Dekalb County	33.781705, -84.285690
Hidden Cove Park	Dekalb County	33.784060, -84.309719
Ira B Melton Park	Dekalb County	33.801861, -84.304497
Kirkwood Urban Forest, Atlanta	Atlanta	33.749274, -84.328702
Legacy Park	Dekalb County	33.768185, -84.276933
Little Mulberry Park	Gwinette County	34.045118, -83.881102
Lullwater Conservation Garden	Atlanta	33.778415, -84.333966
Lullwater Preserve, Emory Univ.	Dekalb County	33.798507, -84.316448
Mary Scott, Decatgur GA	Dekalb County	33.858332, -84.278343
Mason Mill Park (Decatur Waterworks)	Dekalb County	33.804020, -84.304666
Morningside Preserve	Atlanta	33.808761, -84.352798
Panola State Park Stockbridge, GA	Dekalb County	33.625965, -84.171402
Parkwood Park	Atlanta	33.772549, -84.314262
Residence Forest	Atlanta	33.754887, -84.313083
WD Thompson, Decatur	Dekalb County	33.809347, -84.317553
Woodland Gardens, Decatur	Dekalb County	33.786511, -84.303726
Zonolite Park	Atlanta	33.805504, -84.345183
